# Association of habitual diet with skeletal muscle composition in a cross-sectional, population-based imaging study

**DOI:** 10.1186/s12937-025-01222-5

**Published:** 2025-09-23

**Authors:** Susanne Rospleszcz, Theresa Burger, Nuha Shugaa Addin, Lena S. Kiefer, Thierno D. Diallo, Nina Wawro, Christopher L. Schlett, Fabian Bamberg, Annette Peters, Kurt Gedrich, Jakob Linseisen

**Affiliations:** 1 Department of Diagnostic and Interventional Radiology, Faculty of Medicine, University Medical Center Freiburg, University of Freiburg, Killianstr 5a, Freiburg, 79106 Germany; 2 Chair of Epidemiology, Institute for Medical Information Processing, Biometry, and Epidemiology (IBE), Medical Faculty, Ludwig-Maximilians-Universität (LMU), München, Germany; 3 Institute of Epidemiology, Helmholtz Zentrum München, German Research Center for Environmental Health, Neuherberg, Germany; 4 ZIEL - Institute for Food and Health, Research Group Public Health Nutrition, Technical University of Munich, Freising, Germany; 5 Pettenkofer School of Public Health, Munich, Germany; 6 Department of Diagnostic and Interventional Radiology, Eberhard Karls University of Tuebingen, Tuebingen, Germany; 7 Department of Nuclear Medicine and Clinical Molecular Imaging, Eberhard Karls University of Tuebingen, Tuebingen, Germany; 8 German Center for Diabetes Research (DZD), Neuherberg, 85764 Germany; 9 German Centre for Cardiovascular Research (DZHK e.V.), Partner Site Munich Heart Alliance, München, 80802 Germany; 10 Epidemiology, University of Augsburg, University Hospital Augsburg, Augsburg, Germany

**Keywords:** Habitual dietary intake, Skeletal muscle, Adipose tissue, Muscle quality, Sarcopenia, Population-based study

## Abstract

**Background:**

Skeletal muscle health influences overall health and functionality. Nutrition is an important contributor to muscle health, however there is insufficient research on the relation between nutrition and muscle composition, i.e. mass and fatty infiltration, on a population-based level.

**Objective:**

We aimed to investigate the association of habitual dietary intake of energy-providing nutrients (carbohydrates, fat, protein and alcohol) and of essential amino acids with skeletal muscle fat and muscle area derived by magnetic resonance imaging (MRI) in a sample of middle-aged individuals from a population-based cohort.

**Methods:**

We analyzed *N* = 294 individuals (45% women, mean age 56.5 years) from the KORA-MRI study, Southern Germany. Muscle fat (%) and muscle area (cm^2^) were assessed by a multi-echo Dixon sequence on whole-body MRI. Habitual dietary intake was calculated based on repeated 24 h recalls and a food frequency questionnaire. Correlation analyses and adjusted regression models were calculated.

**Results:**

Alcohol intake was associated with increased skeletal muscle fat, particularly in men (β = 0.28%, 95% confidence interval [0.10%,0.45%]; *p* = 0.002) per percent of total energy intake). Protein intake was tentatively associated with lower muscle fat (β=-0.33% [-0.68%,0.01%]; *p* = 0.052). Accounting for overall protein and energy, specific essential amino acids were tentatively associated with lower muscle fat, e.g. leucine (β=-0.63%, [-1.27%,0.01%]; *p* = 0.054).

**Conclusion:**

In middle-aged adults, habitual alcohol and protein intake are associated with fatty infiltration of skeletal muscle. Individualized diet adaptations might improve muscle composition and function.

**Supplementary Information:**

The online version contains supplementary material available at 10.1186/s12937-025-01222-5.

## Introduction

Muscle health, including muscle strength, endurance, and metabolic activity, is a fundamental aspect of overall health, functionality, and longevity. Skeletal muscle contributes to maintaining strength, balance and mobility, resulting in improved functionality in daily activities and reducing the risk of falls and injuries [1]. Moreover, adequate skeletal muscle mass helps to regulate blood glucose levels, enhances insulin sensitivity and promotes healthy lipid profiles [2].

Muscle composition parameters, assessed by medical imaging, such as computed tomography or magnetic resonance (MRI), improve our understanding of the role of skeletal muscle in metabolic health [3, 4]. In particular, ectopic fat infiltration of skeletal muscle (myosteatosis) is an essential marker of muscle quality, and increased muscle fat is associated with decreased muscle function [5], deteriorated mobility [6] and metabolic impairment [7]. In a large retrospective outpatient study, imaging-derived myosteatosis was the strongest body composition parameter for mortality in asymptomatic patients [8].

Nutrition is an important contributor to muscle composition, as it provides the essential nutrients necessary for muscle protein synthesis and maintenance, influencing both muscle mass and muscle strength [9]. Recognizing the tight link between nutrition and muscle composition and function, the Global Leadership Initiative on Malnutrition has identified loss of muscle mass and muscle strength as a supportive measure in the definition of malnutrition [10]. The association between inadequate nutrient intake and myosteatosis is particularly visible in older individuals, since ectopic fat infiltration exacerbates age-related anabolic resistance, causing increased protein demands to achieve the same rate of muscle protein synthesis. Thus, associations between inadequate nutrient intake and muscle fat, mass and strength have been shown [11], often in the context of sarcopenia, i.e. loss of muscle function, strength and mass, particularly in older individuals [12].

Essential amino acids, which cannot be synthesized by the human body have been suggested to play a role in improving and maintaining muscle mass and strength. In recent meta-analyses on supplements of leucine or branched-chain amino acids (BCAA), supplements improved muscle parameters and physical performance in older adults with sarcopenia [13, 14]. However, the association of habitual intake of essential amino acids with muscle quality in younger or middle-aged individuals has not been comprehensively studied.

Given the importance of maintaining muscle health for healthy aging, it is especially relevant to study the impact of nutrition on muscle composition on a population-based level. Moreover, known differences in diet and muscle composition between men and women require the investigation of potential sex-specific effects. We thus aim to investigate the overall and sex-specific association of habitual dietary intake of energy-providing nutrients (carbohydrates, fat, protein, alcohol) and essential amino acids (phenylalanine, valine, tryptophan, threonine, isoleucine, methionine, histidine, leucine, lysine) on muscle fat and muscle area, as derived by MRI, in a sample from a population-based cohort.

## Methods

### Study sample

The analysis is based on the KORA-MRI study, which is a subsample of the KORA-FF4 study (KORA: “Cooperative Health Research in the Region of Augsburg”). KORA-FF4 (enrolled in 2013–2014, *N* = 2279) is the second follow-up of the baseline KORA-S4 survey (enrolled in 1999–2001, *N* = 4261). General setup and sampling scheme of this population-based survey are detailed elsewhere [15]. KORA-MRI consists of *N* = 400 individuals who underwent whole-body magnetic resonance imaging, as previously described [16]. Briefly, KORA-MRI focused on the evaluation of systemic metabolic effects of glycemic impairment. Inclusion criteria were willingness to undergo MRI, and availability of data on glycemia (normoglycemia, prediabetes, diabetes). Exclusion criteria were any contraindications to MRI (claustrophobia, metal parts inside the body, allergy to contrast agent), age older than 73 years, and a known history of cardiovascular disease (myocardial infarction, stroke, revascularization). Before the MRI examination, all participants underwent a standardized face-to-face interview, a physical examination and a blood draw at the central KORA study center.

The study was approved by the ethics committee of Ludwig-Maximilians-University Munich (498 − 12) and the Bavarian Chamber of Physicians (FF4: EC No. 06068); and was performed according to the Declaration of Helsinki, including written informed consent of all participants.

For the current study, a total of 106 individuals had to be excluded from analysis. One participant retroactively withdrew consent for data usage. Then, 25 individuals had to be excluded due to missing or incomplete MRI data. In addition, 80 further individuals had incomplete data on habitual dietary intake. Thus, the final sample consisted of *N* = 294 participants.

### Outcome assessment: MRI-derived muscle fat and muscle area

The MRI examinations were conducted using a 3-Tesla Magnetom Skyra (Siemens Healthineers, Erlangen, Germany) with a whole-body coil system within three months after the original visit at the study center. Detailed information on the whole-body protocol is described elsewhere [16]. Images of four skeletal muscle groups were obtained on a T2*-corrected, multi-echo 3D-gradient Dixon sequence during a single breath hold [17]. Manual segmentation of each muscle compartment was then performed by two independent readers using standardized anatomical landmarks at the lower endplate of the L3 vertebra, as previously described [18].

For subsequent statistical analysis, skeletal muscle area in cm^2^ was defined as the sum of the left and right areas of M. psoas major, M. quadratus lumborum, autochthonous back muscles, and M. rectus abdominis. Skeletal muscle fat in % was defined as the average of the fat fraction of these muscles, weighted by their respective area.

### Exposure assessment: habitual dietary intake

Dietary intake was recorded using a blended approach, combining a self-administered food frequency questionnaire (FFQ) and up to three repeated 24-hour recalls [19] within three months after the first visit at the study center (Supplementary Text 1). Based on these data, the probability of consuming a particular food was calculated. Then, the food quantity typically consumed on days with consumption was estimated based on the Bavarian Food Consumption Survey II [20]. Habitual diet was then calculated by multiplying consumption probability and amount. Food nutrient contents were taken from the German Nutrient Database “Bundeslebensmittelschlüssel”, version 3.02 [21]. For the present analysis, data of interest were total habitual energy intake, habitual intake of energy-providing nutrients (carbohydrates, fat, protein, alcohol) and habitual intake of essential amino acids (phenylalanine, valine, tryptophan, threonine, isoleucine, methionine, histidine, leucine, lysine), as well as intake of branched-chain amino acids (BCAA, i.e. valine, leucine and isoleucine).

### Covariate assessment

Anthropometric measures were assessed by standardized procedures with calibrated instruments as previously described [22]. Smoking behavior was based on self-report and categorized into never, former and current smoker. Physical activity was categorized according to self-reported sports activities as regularly 2 h/week or more, regularly up to 1 h/week, sporadic, and no to almost no physical activity. Blood pressure was measured three times with 3-minute intervals in between. The mean value of the second and third measurement was used as the final value. Hypertension was defined as systolic/diastolic blood pressure > 140/90 mmHg or intake of antihypertensive medication under the awareness of having hypertension. Glycemia was categorized as normoglycemia, prediabetes or diabetes, either based on prior physician report, or based on an Oral Glucose Tolerance Test according to WHO criteria. Lipid profile, inflammation, liver enzymes, and markers of kidney function were measured by standard laboratory measures as described previously [23].

### Protein quality scoring

To assess quality of protein intake, we calculated individual recommended intake of each essential amino acid by scaling the respective WHO recommended daily allowance (RDA) [24] to individual body weight. Accordingly, methionine intake was subsumed with cysteine intake, and phenylalanine intake subsumed with tyrosine intake. We then divided actual individual intake by the individual recommendation to calculate percentage of recommendation attained. Mean, minimum and maximum value of percentage of recommendation attained are described for the whole sample as well as sex-stratified.

### Statistical analysis

Clinical characteristics, MRI-derived muscle fat and muscle area and dietary data of participants are presented as arithmetic mean with standard deviation for continuous data and counts with percentages for categorical data. Differences between women and men were assessed by t-test or X^2^ test, respectively. Characteristics of participants in the final sample (*N* = 294) and individuals excluded from the final analysis due to missing MRI or dietary data (*N* = 104) were compared with t-test or X^2^ test, respectively.

Correlations between variables (muscle fat and muscle area, diet and muscle fat, diet and muscle area) were visualized by sex-stratified scatter plots and quantified by Spearman’s correlation coefficients. All correlations were unadjusted.

Linear regression models with outcome muscle fat or muscle area and exposure habitual diet were calculated to obtain β estimates and corresponding 95% confidence intervals (CI). Generalized additive models (GAM) with a cubic spline basis and up to 5 knots, allowing for non-linear nutrient intake associations, showed no substantial improvement over linear models based on variance explained and AIC, and were therefore not pursued further. Exposures of habitual diet were scaled to 1% of total energy intake, assuming an energy value of 4 kcal/g for carbohydrates, 9 kcal/g for fat, 4 kcal/g for protein and 7 kcal/g for alcohol. Models were calculated for the whole sample as well as sex-stratified. Additionally, multiplicative interaction between sex and nutrient intake was formally tested. Models were adjusted for potential confounding variables age, sex (for the whole sample), BMI, physical activity (4 categories), and glycemia (normoglycemia/prediabetes/diabetes), which were chosen a-priori based on prior knowledge. In two sensitivity analyses, models were additionally adjusted for smoking, and BMI was replaced by waist circumference. For exposure amino acids, values were scaled to 1% of total protein intake by standardizing the ratio of amino acids in mg/d to total protein intake in mg/d, and the models were additionally adjusted for total energy intake.

All statistical analyses were done with R version 4.1.1. We consider *p*-values < 0.05 and < 0.1 to denote statistical and tentative statistical significance. *P*-values were not corrected for multiple testing.

## Results

### Study sample

The final sample included *N* = 294 participants (132 women) with a mean age of 56.5 ± 9.0 years, a mean BMI of 27.8 ± 4.8 kg/m2 and a prevalence of diabetes of 12.2% (Table 1). Compared to the final sample, individuals that had been excluded due to missing MRI data or missing habitual diet data had larger body size as indicated by higher weight, BMI, waist and hip circumference and their ratio (Supplementary Table 1). Moreover, excluded individuals had higher HbA1c and triglyceride levels, but there were no significant differences in age, sex, blood pressure or smoking behavior (Supplementary Table 1).

**Table 1:** Clinical characteristics of the study sample

	Whole sample	Women	Men	*p*-value
	*N* = 294	*N* = 132 (44.9%)	*N* = 162 (55.1%)	
Demographics				
Age, years	56.5 ± 9.0	56.2 ± 8.7	56.6 ± 9.3	0.715
Anthropometrics				
Height, cm	171.2 ± 9.8	163.3 ± 6.3	177.7 ± 6.9	< 0.001
Weight, kg	81.6 ± 15.9	73.3 ± 15.0	88.4 ± 13.3	< 0.001
BMI, kg/m^2^	27.8 ± 4.8	27.5 ± 5.5	28.0 ± 4.2	0.394
Waist circumference, cm	97.5 ± 14.0	91.4 ± 14.1	102.4 ± 11.9	< 0.001
Hip circumference, cm	106.5 ± 8.9	106.5 ± 10.6	106.4 ± 7.2	0.950
Waist-to-Hip Ratio	0.914 ± 0.089	0.856 ± 0.077	0.960 ± 0.069	< 0.001
Lifestyle factors				
Smoking behaviour				
never smoker	109 (37.1%)	54 (40.9%)	55 (34.0%)	0.299
former smoker	128 (43.5%)	51 (38.6%)	77 (47.5%)	
current smoker	57 (19.4%)	27 (20.5%)	30 (18.5%)	
Physical activity				0.05
regularly 2 h/w or more	87 (29.6%)	40 (30.3%)	47 (29.0%)	
regularly 1 h/w	93 (31.6%)	49 (37.1%)	44 (27.2%)	
sporadic	41 (13.9%)	20 (15.2%)	21 (13.0%)	
inactive	73 (24.8%)	23 (17.4%)	50 (30.9%)	
Blood pressure				
Systolic Blood Pressure, mmHg	120.2 ± 16.4	113.3 ± 14.6	125.8 ± 15.6	< 0.001
Diastolic Blood Pressure, mmHg	75.0 ± 10.0	72.1 ± 8.8	77.3 ± 10.3	< 0.001
Hypertension	104 (35.4%)	37 (28.0%)	67 (41.4%)	0.024
Antihypertensive medication	80 (27.2%)	35 (26.5%)	45 (27.8%)	0.912
Diabetes-related				
Glycemia				
Normogylcemia	185 (62.9%)	93 (70.5%)	92 (56.8%)	0.051
Prediabetes	73 (24.8%)	27 (20.5%)	46 (28.4%)	
Diabetes	36 (12.2%)	12 (9.1%)	24 (14.8%)	
HbA1c, %	5.5 ± 0.6	5.6 ± 0.6	5.5 ± 0.6	0.638
Fasting glucose, mg/dL	103.1 ± 17.6	98.9 ± 16.7	106.5 ± 17.6	< 0.001
2-h glucose, mg/dL^#^	111.9 ± 41.2	107.1 ± 35.8	116.0 ± 44.9	0.077
Fasting insulin, µU/mL	11.1 ± 7.2	10.0 ± 5.8	11.9 ± 8.1	0.022
2-h insulin, µU/mL^#^	64.3 ± 66.4	58.4 ± 48.5	69.2 ± 78.1	0.185
Glucose-lowering medication	23 (7.8%)	10 (7.6%)	13 (8.0%)	1
Lipid profile				
Total Cholesterol, mg/dL	217.7 ± 36.6	219.7 ± 35.6	216.1 ± 37.4	0.410
HDL Cholesterol, mg/dL	62.9 ± 17.9	70.7 ± 17.8	56.6 ± 15.2	< 0.001
LDL Cholesterol, mg/dL	139.4 ± 33.6	136.6 ± 33.2	141.8 ± 33.8	0.185
Triglycerides, mg/dL	126.2 ± 79.5	103.5 ± 47.2	144.7 ± 94.4	< 0.001
Lipid-lowering medication	33 (11.2%)	16 (12.1%)	17 (10.5%)	0.800
Inflammation				
Uric Acid, mg/dL	5.6 ± 1.5	4.7 ± 1.1	6.4 ± 1.4	< 0.001
hsCRP, mg/L	2.4 ± 3.5	2.6 ± 3.8	2.2 ± 3.2	0.428
Liver and kidney markers				
GGT, µkat/l	0.64 ± 0.65	0.49 ± 0.54	0.76 ± 0.71	< 0.001
ASAT, µkat/l	0.42 ± 0.23	0.38 ± 0.19	0.46 ± 0.25	0.005
ALT, µkat/l	0.50 ± 0.29	0.42 ± 0.26	0.58 ± 0.28	< 0.001
Creatinine, mg/dL	0.88 ± 0.15	0.78 ± 0.12	0.96 ± 0.13	< 0.001
eGFR, mL/min/1.73m^2^	86.6 ± 13.1	85.7 ± 13.4	87.3 ± 12.9	0.303

Data are means and standard deviation for continuous variables, and counts and percentages for categorical values. *P*-values for women vs. men from t-test and Χ^2^ test, respectively

^#^based on *n* = 363 with Oral Glucose Tolerance Test data

**Fig. 1:**
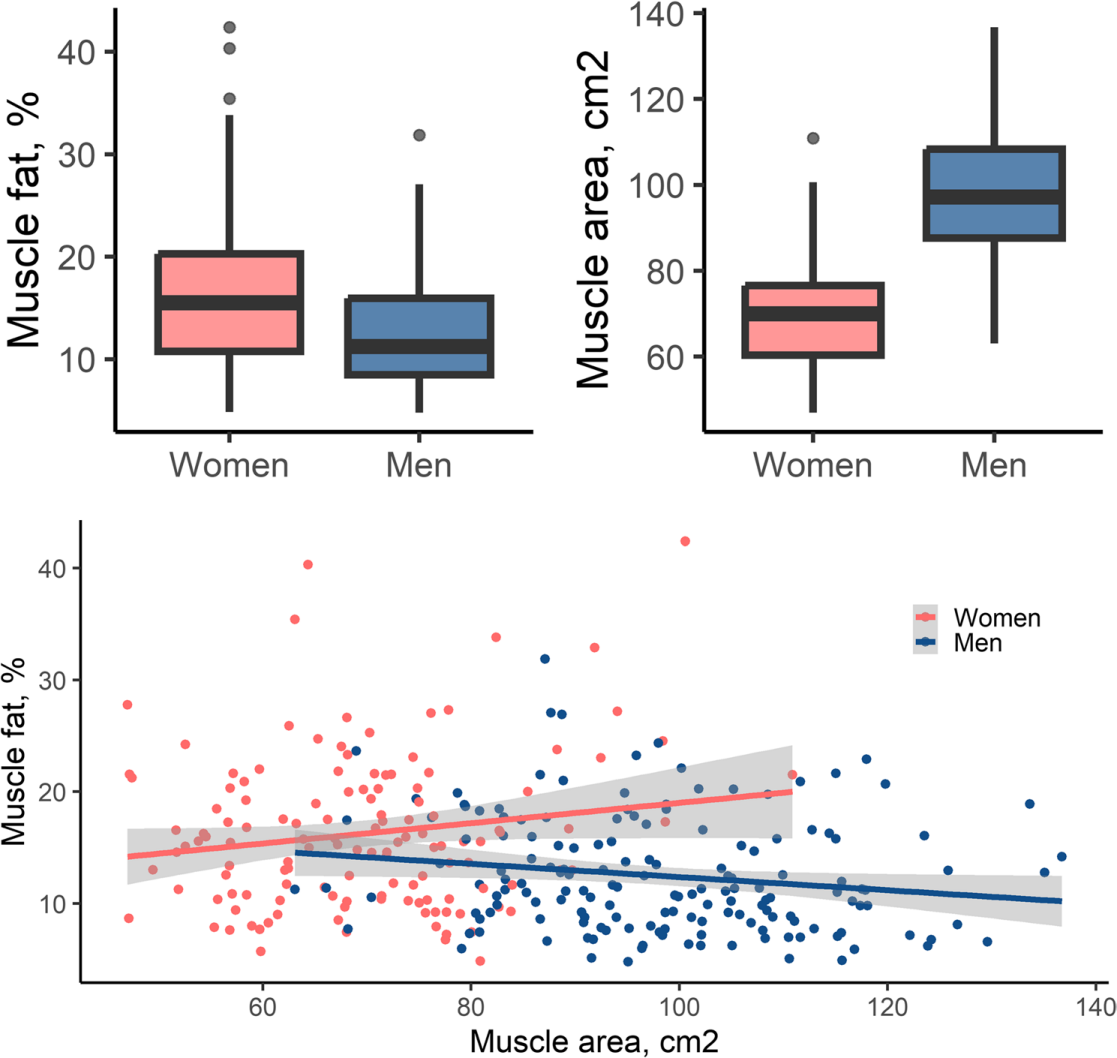
Distribution of MRI-derived muscle fat and area in the sample

In the final sample, average MRI-derived skeletal muscle fat was 16.3 ± 6.9% in women and 12.5 ± 5.2% in men (Fig. 1). Average MRI-derived muscle area was 69.7 ± 12.1cm^2^ in women and 98.1 ± 14.6cm^2^ in men (Fig. 1). In women, muscle fat and muscle area were not correlated (*r* = 0.05, *p* = 0.591), whereas in men, muscle fat and muscle area were negatively correlated (*r*=−0.19, *p* = 0.014, Fig. 1, *p*-value for sex interaction: 0.005).

Mean total energy intake was 1555 ± 296 kcal/d in women and 2063 ± 352 kcal/d in men (Table 2). On average, 41.7% of energy intake came from carbohydrates, 38.0% from fat, 15.4% from protein and 4.2% from alcohol, with significant differences between women and men (Table 2). Also, habitual intake of essential amino acids differed substantially between women and men (Table 2).

**Table 2:** Habitual diet of the study sample: total energy intake, and intake of energy-providing nutrients and essential amino acids

		Whole sample	Women	Men	*p*-value
		*N* = 294	*N* = 132	*N* = 162	
Total energy intake, kcal/d		1835 ± 414	1555 ± 296	2063 ± 352	< 0.001
Energy-providing nutrients					
Carbohydrates	g/d	192.0 ± 50.0	166.2 ± 38.8	213.1 ± 48.3	< 0.001
	% of total energy intake	41.7 ± 4.1	42.5 ± 3.7	41.1 ± 4.3	0.003
Fat	g/d	77.0 ± 16.7	66.6 ± 13.2	85.4 ± 14.3	< 0.001
	% of total energy intake	38.0 ± 3.5	38.7 ± 3.4	37.4 ± 3.5	0.003
Protein	g/d	69.9 ± 15.1	61.5 ± 12.5	76.7 ± 13.5	< 0.001
	% of total energy intake	15.4 ± 1.7	15.9 ± 1.7	14.9 ± 1.5	< 0.001
Alcohol	g/d	11.6 ± 10.9	4.5 ± 4.7	17.4 ± 11.1	< 0.001
	% of total energy intake	4.2 ± 3.6	2.1 ± 2.2	5.9 ± 3.6	< 0.001
Essential Amino Acids					
Phenylalanine	mg/d	3062.7 ± 662.9	2730.0 ± 571.7	3333.8 ± 607.6	< 0.001
	% of protein	4.4 ± 0.1	4.4 ± 0.1	4.3 ± 0.1	< 0.001
Valine	mg/d	3862.1 ± 841.1	3421.7 ± 715.0	4221.0 ± 763.5	< 0.001
	% of protein	5.5 ± 0.1	5.6 ± 0.1	5.5 ± 0.1	< 0.001
Tryptophan	mg/d	812.9 ± 179.4	711.8 ± 149.7	895.3 ± 158.4	< 0.001
	% of protein	1.2 ± 0.0	1.2 ± 0.0	1.2 ± 0.0	< 0.001
Threonine	mg/d	2843.2 ± 624.5	2495.3 ± 511.9	3126.6 ± 562.6	< 0.001
	% of protein	4.1 ± 0.1	4.1 ± 0.1	4.1 ± 0.1	0.116
Isoleucine	mg/d	3293.6 ± 712.3	2918.3 ± 601.2	3599.3 ± 647.8	< 0.001
	% of protein	4.7 ± 0.1	4.7 ± 0.1	4.7 ± 0.1	< 0.001
Methionine	mg/d	1566.6 ± 352.5	1385.8 ± 299.2	1713.9 ± 323.4	< 0.001
	% of protein	2.2 ± 0.1	2.2 ± 0.1	2.2 ± 0.1	0.043
Histidine	mg/d	1989.3 ± 438.7	1712.1 ± 334.0	2215.2 ± 380.9	< 0.001
	% of protein	2.8 ± 0.1	2.8 ± 0.1	2.9 ± 0.1	< 0.001
Leucine	mg/d	5469.6 ± 1197.4	4890.1 ± 1048.7	5941.7 ± 1102.1	< 0.001
	% of protein	7.8 ± 0.2	7.9 ± 0.2	7.7 ± 0.2	< 0.001
Lysine	mg/d	4744.7 ± 1061.3	4185.4 ± 894.4	5200.4 ± 966.1	< 0.001
	% of protein	6.8 ± 0.3	6.8 ± 0.3	6.8 ± 0.3	0.334
BCAA	mg/d	12,625 ± 2747.5	11,230 ± 2361.9	13,762 ± 2510.5	< 0.001
	% of protein	18.1 ± 0.3	18.2 ± 0.3	17.9 ± 0.3	< 0.001

*P*-values for women vs. men from t-test

*BCAA* branched-chain amino acids (valine, isoleucine, leucine), *% of protein* % of total protein intake

### Association of energy-providing nutrient intake with muscle fat and muscle area

**Fig. 2:**
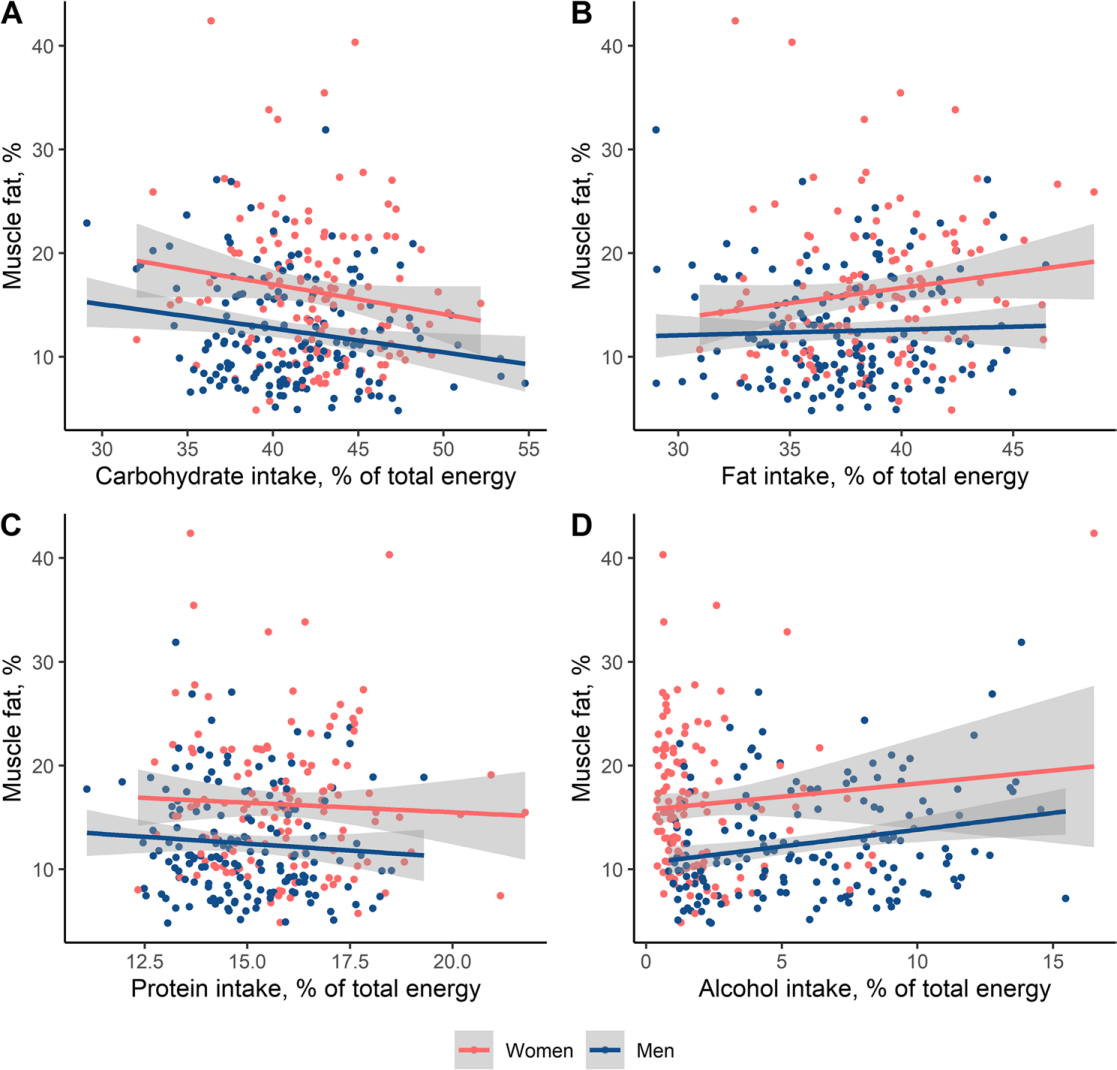
Correlation of intake of energy-providing nutrients with muscle fat

Carbohydrate intake, as % of total energy intake, was negatively correlated with muscle fat in both women (*r*=−0.15, *p* = 0.078) and men (*r*=−0.14, *p* = 0.075, Fig. 2A). However, after adjustment for potential confounding variables age, sex, BMI, physical activity and glycemia, carbohydrate intake was not associated with muscle fat in the overall sample, in women, or men (Table 3). Carbohydrate intake was not correlated or associated with muscle area (Supplementary Fig. 1 A, Table 3).

**Table 3:** Association of habitual energy-providing nutrient intake with MRI-derived muscle fat and area

	Outcome Muscle Fat, %			Outcome Muscle Area, cm^2^		
	β	95%-CI	*p*-value	β	95%-CI	*p*-value
Carbohydrates, per 1% of total energy intake						
All	−0.06	[−0.20, 0.07]	0.356	−0.11	[−0.47, 0.24]	0.520
Women	−0.01	[−0.26, 0.23]	0.911	−0.05	[−0.56, 0.47]	0.859
Men	−0.11	[−0.27, 0.05]	0.161	−0.18	[−0.67, 0.31]	0.465
Fat, per 1% of total energy intake						
All	−0.10	[−0.27, 0.06]	0.209	0.11	[−0.30, 0.53]	0.589
Women	−0.13	[−0.41, 0.14]	0.335	−0.40	[−0.96, 0.17]	0.167
Men	−0.09	[−0.28, 0.11]	0.380	0.44	[−0.16, 1.04]	0.153
Protein, per 1% of total energy intake						
All	−0.34	[−0.69, 0.00]	0.052	−0.11	[−1.00, 0.77]	0.801
Women	−0.23	[−0.78, 0.31]	0.403	−0.21	[−1.35, 0.92]	0.710
Men	−0.31	[−0.76, 0.13]	0.169	0.16	[−1.22, 1.53]	0.823
Alcohol, per 1% of total energy intake						
All	0.31	[0.14, 0.48]	< 0.001	0.08	[−0.37, 0.53]	0.721
Women^#^	−0.01	[−0.49, 0.47]	0.971	0.61	[−0.41, 1.63]	0.240
Men	0.28	[0.10, 0.45]	0.002	−0.17	[−0.73, 0.39]	0.545

The table shows results of a linear regression model with exposure habitual energy-providing nutrient intake as % of total energy intake, and outcome muscle fat or area. Models were adjusted for age, sex (for the whole sample), BMI, physical activity (4 categories), and glycemia (normoglycemia/prediabetes/diabetes)

^#^based on *N* = 131 after excluding one outlier. There was no statistically significant formal interaction of sex with intake of any of the four nutrients

Fat intake, as % of total energy intake, was positively correlated with muscle fat in women (*r* = 0.20, *p* = 0.019), but not in men (*r* = 0.04, *p* = 0.624, Fig. 2B). After adjustment, there was no association with muscle fat in the overall sample, in women, or men (Table 3). Fat intake was not correlated or associated with muscle area (Supplementary Fig. 1B, Table 3).

Protein intake, as % of total energy intake, was not correlated with muscle fat in women (*r*=−0.02, *p* = 0.787) nor men (*r*=−0.09, *p* = 0.249, Fig. 2C). However, after adjustment for potential confounding variables, increased protein intake was tentatively associated with lower muscle fat in the overall sample (β=−0.34%, 95%-CI [−0.69%, 0.00%], *p* = 0.052), though the associations were attenuated in the sex-stratified analyses (Table 3). To contextualize the effect sizes, age was associated with an increase of 0.34% [0.28%, 0.40%] in muscle fat per year, and BMI with an increase of 0.38% [0.25%, 0.50%] per unit kg/m^2^. Effect estimates for all adjustment variables are presented in Supplementary Table 3. Protein intake was correlated with muscle area (women: *r* = 0.16, *p* = 0.072, men: *r* = 0.14, *p* = 0.086, Supplementary Fig. 1 C), but after adjustment for confounders, no association remained (Table 3).

Alcohol intake, as % of total energy intake, was not correlated with muscle fat in women (*r*=−0.11, *p* = 0.113), but in men (*r* = 0.23, *p* = 0.004, Fig. 2D). After adjustment for potential confounding variables, increased alcohol intake was associated with higher muscle fat in the overall sample (β = 0.31%, 95%-CI [0.14%, 0.48%], *p* < 0.001) and in men (Table 3). In women, there was no association after exclusion of an outlier value that had shown high influence and leverage in model diagnostics. To contextualize the effect sizes, age was associated with an increase of 0.34% [0.27%, 0.40%] in muscle fat per year, and BMI with an increase of 0.38% [0.26%, 0.50%] per unit kg/m^2^. Alcohol intake was not correlated or associated with muscle area (Supplementary Fig. 1D, Table 3).

In sensitivity analyses with additional adjustment for smoking behavior, or replacing BMI by waist circumference, results remained stable (Supplementary Table 4).

### Protein quality scoring

Recommended intake of essential amino acids was calculated for each individual based on WHO RDA and individual weight, and percentage of individual recommendation attained was computed. Recommended intake was on average substantially exceeded for all essential amino acids (Supplementary Fig. 3), ranging from 176.3% (± SD 45.2%) for leucine intake to 278.5% (± SD 72.1%) for phenylalanine and tyrosine (Supplementary Table 5). Although variation in percentage of individual recommendation attained was higher in women compared to men for every amino acid, differences in mean values were only statistically significant for histidine intake (241.1%± 62.3% in women compared to 255.1%± 53.9% in men, *p* = 0.040, Supplementary Table 5).

### Association of essential amino acid intake with muscle fat and muscle area

**Fig. 3:**
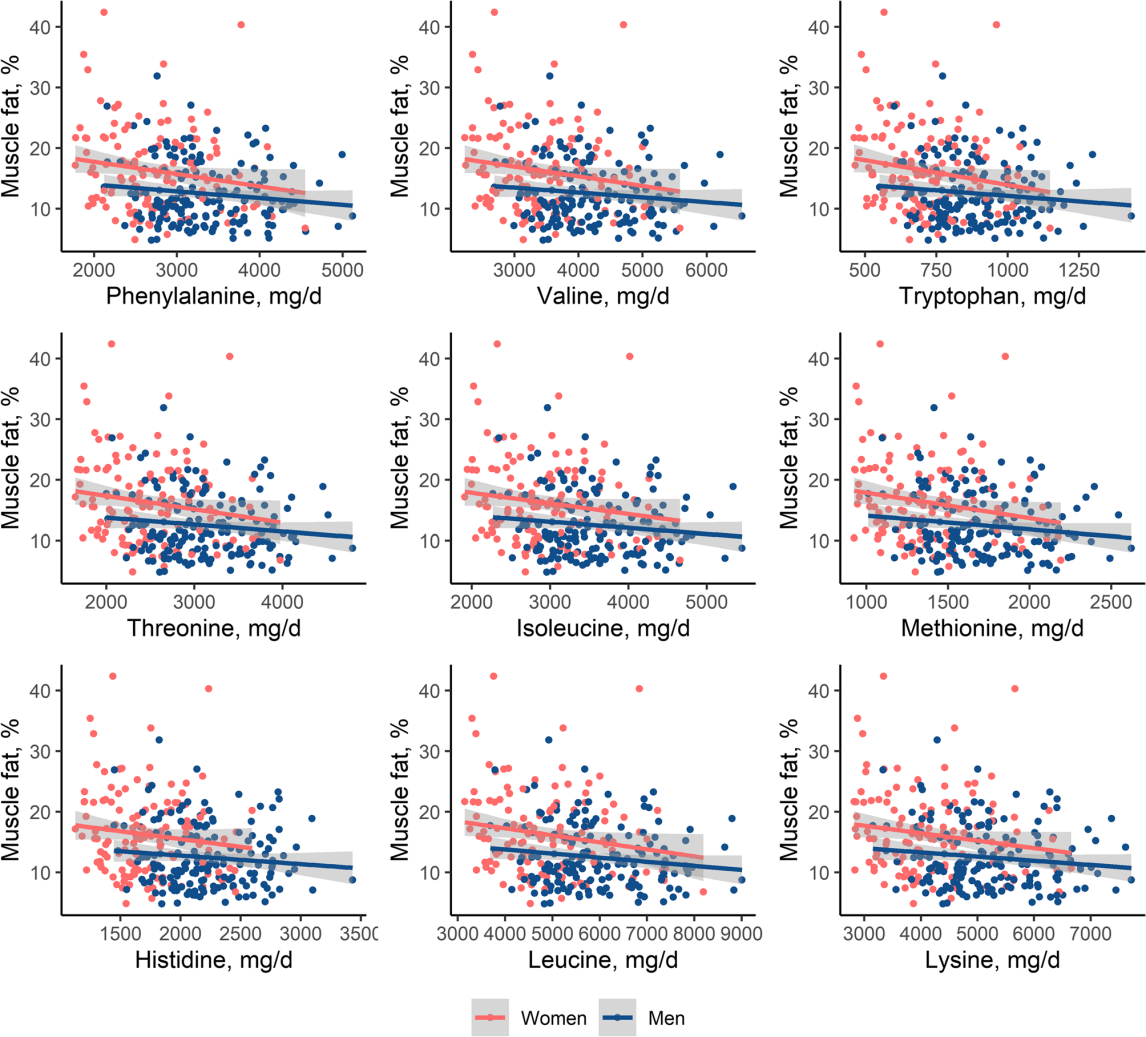
Correlation of habitual intake of essential amino acids with muscle fat

In both women and men, habitual intake of essential amino acids phenylalanine, valine, tryptophan, threonine, isoleucine, methionine, histidine, leucine, lysine was negatively correlated with muscle fat to varying degrees (Fig. 3). In women, strongest correlation was observed for methionine (*r*=−0.19, *p* = 0.032) and weakest correlation for histidine (*r*=−0.13, *p* = 0.136). In men, strongest correlation was observed for leucine (*r*=−0.11, *p* = 0.174) and weakest correlation for histidine (*r*=−0.07, *p* = 0.369). After scaling amino acids to standardized % of total protein intake, and adjusting for age, sex, BMI, physical activity, glycemia and total energy intake, there were tentative associations of increased methionine intake (β=−0.50%, 95%-CI [−1.09%, 0.09%], *p* = 0.096) and increased leucine (β=−0.63%, 95%-CI [−1.27%, 0.01%], *p* = 0.054) with decreased muscle fat in the whole sample. Moreover, increased valine intake was tentatively associated (β=−0.80%, 95%-CI [−1.69%, 0.09%], *p* = 0.078), and increased tryptophan intake was significantly associated with decreased muscle fat in women (β=−1.09%, 95%-CI [−1.99%, −0.19%], *p* = 0.018, Table 4).

**Table 4:** Association of habitual intake of essential amino acids with MRI-derived muscle fat and area

	Outcome Muscle Fat, %			Outcome Muscle Area, cm^2^		
	β	95%-CI	*p*-value	β	95%-CI	*p*-value
Phenylalanine, per SD (% of total protein intake)						
All	−0.50	[−1.17, 0.18]	0.151	−0.59	[−2.33, 1.15]	0.503
Women	−0.84	[−2.03, 0.34]	0.159	−1.43	[−3.88, 1.03]	0.252
Men	−0.30	[−1.11, 0.51]	0.463	−0.19	[−2.69, 2.31]	0.880
Valine, per SD (% of total protein intake)						
All	−0.36	[−0.93, 0.21]	0.218	−0.61	[−2.07, 0.85]	0.415
Women	−0.80	[−1.69, 0.09]	0.078	−1.15	[−3.00, 0.71]	0.225
Men	0.04	[−0.69, 0.78]	0.906	0.28	[−2.00, 2.55]	0.810
Tryptophan, per SD (% of total protein intake)						
All	−0.24	[−0.79, 0.30]	0.381*	−0.40	[−1.79, 0.99]	0.572
Women	−1.09	[−1.99, −0.19]	0.018	0.56	[−1.35, 2.47]	0.564
Men	0.46	[−0.19, 1.12]	0.162	−0.80	[−2.82, 1.22]	0.436
Threonine, per SD (% of total protein intake)						
All	0.14	[−0.42, 0.71]	0.616	0.86	[−0.58, 2.31]	0.241
Women	0.20	[−0.81, 1.21]	0.694	1.19	[−0.89 3.28]	0.260
Men	0.17	[−0.49, 0.82]	0.620	0.93	[−1.09, 2.96]	0.365
Isoleucine, per SD (% of total protein intake)						
All	−0.14	[−0.73, 0.44]	0.628	0.07	[−1.42, 1.56]	0.931
Women	−0.17	[−1.13, 0.79]	0.727	0.11	[−1.87, 2.10]	0.910
Men	−0.22	[−0.95, 0.51]	0.558	0.19	[−2.06, 2.44]	0.868
Methionine, per SD (% of total protein intake)						
All	−0.50	[−1.09, 0.09]	0.096	0.46	[−1.06, 1.98]	0.553
Women	−0.53	[−1.53, 0.46]	0.290	−0.51	[−2.57, 1.56]	0.627
Men	−0.33	[−1.05, 0.39]	0.367	1.58	[−0.64, 3.80]	0.161
Histidine, per SD (% of total protein intake)						
All	0.35	[−0.37, 1.06]	0.340	0.95	[−0.87, 2.77]	0.303
Women	0.07	[−1.28, 1.42]	0.920	2.54	[−0.22, 5.30]	0.071
Men	0.20	[−0.62, 1.02]	0.630	0.09	[−2.43, 2.61]	0.945
Leucine, per SD (% of total protein intake)						
All	−0.63	[−1.27, 0.01]	0.054	−0.04	[−1.68, 1.61]	0.965
Women	−0.41	[−1.42, 0.60]	0.423	−1.46	[−3.55, 0.62]	0.167
Men	−0.64	[−1.46, 0.17]	0.121	1.65	[−0.86, 4.17]	0.196
Lysine, per SD (% of total protein intake)						
All	−0.20	[−0.79, 0.40]	0.519	0.99	[−0.54, 2.51]	0.204
Women	−0.03	[−1.01, 0.95]	0.950	0.12	[−1.91, 2.15]	0.908
Men	−0.22	[−0.96, 0.52]	0.550	0.22	[−0.08, 4.43]	0.059
BCAA, per SD (% of total protein intake)						
All	−0.50	[−1.11, 0.12]	0.113	−0.19	[−1.76, 1.39]	0.813
Women	−0.55	[−1.54, 0.45]	0.278	−1.18	[−3.23, 0.87]	0.257
Men	−0.39	[−1.15, 0.38]	0.321	1.00	[−1.36, 3.38]	0.404

The table shows results of a linear regression model with exposure habitual intake of essential amino acids as standardized % of total protein intake, and outcome muscle fat or area. Models were adjusted for age, sex (for the whole sample), BMI, physical activity (4 categories), glycemia (normoglycemia/prediabetes/diabetes) and total energy intake

*BCAA* branched-chain amino acids (valine, leucine, isoleucine)

*statistically significant formal sex interaction

In both women and men, habitual intake of essential amino acids was positively correlated with muscle area to varying degrees (Supplementary Fig. 2). In women, strongest correlation was observed for histidine (*r* = 0.16, *p* = 0.074) and weakest correlation for phenylalanine (*r* = 0.09, *p* = 0.318). In men, strongest correlation was observed for lysine (*r* = 0.21, *p* = 0.006) and weakest correlation for phenylalanine (*r* = 0.15, *p* = 0.054). After adjustment for potential confounding variables, there were tentative associations of increased histidine intake with increased muscle area in women (β = 2.54cm^2^, 95%-CI [−0.22cm^2^, 5.30cm^2^], *p* = 0.071) and of increased lysine intake with increased muscle area in men (β = 0.22cm^2^, 95%-CI [−0.08cm^2^, 4.43cm^2^], *p* = 0.059, Table 4).

## Discussion

In this analysis of middle-aged adults in a sample from a population-based cohort, we assessed the association between habitual dietary intake and imaging-derived muscle fat and muscle area. Results showed that increased alcohol intake was associated with increased muscle fat, particularly in men, whereas increased protein intake was associated with lower muscle fat. Moreover, the amino acid composition of consumed proteins was relevant - in particular, increased methionine and leucine intake, and in women increased tryptophan intake were associated with decreased muscle fat.

Myosteatosis has emerged as a relevant metric of muscle quality, independent from sarcopenia. Since age-related decline in muscle strength cannot be explained by loss of muscle mass alone, muscle quality is hypothesized to play a major role in maintaining muscle function and mobility [25, 26]. A correlation of increased fatty infiltration with decreased muscle function has already been shown [27]; moreover, myosteatosis is strongly related to unfavorable cardiometabolic outcomes [28]. It is therefore relevant to study potentially modifiable factors of muscle composition, such as nutrition.

In our study, we found no association of habitual carbohydrate or fat intake with muscle fat or muscle area. This is in contrast to a recent study from Korea, that reported a significant association of higher carbohydrate intake with higher muscle fat in *n* = 35 individuals with obesity; however these analyses were not corrected for total energy intake [29]. A British study on = 391 individuals reported no significant correlation between carbohydrate or fat intake (as % of total energy intake) with total body fat, fat mass or lean mass. There was, however, a positive correlation between protein intake and lean mass [30].

### Alcohol and muscle composition

Evidence for the effects of alcohol consumption on muscle fatty infiltration on a population-wide level is scarce. In a study on patients with acute pancreatitis and control individuals, high alcohol consumption was associated with increased skeletal muscle fat [31]. In our data, we found an association of increased alcohol intake with increased muscle fat with an effect size comparable to one year of aging, but smaller than one unit increase in BMI. This effect was only visible in men. Animal studies have shown that alcohol consumption has sex-specific effects on muscle contractility in mice. Though muscle function was decreased after acute alcohol consumption in both female and male mice, muscles in female mice completely recovered after 24 h, whereas muscles in male mice did not [32].

Although it is known that pharmacokinetics of alcohol differ between women and men, evidence from animal studies points towards an increased susceptibility of female mice to accumulate adipose tissue as a response to alcohol injury [32]. Thus, there is no straightforward biological explanation why alcohol would act on muscle fat in men, but not in women. We therefore hypothesize our findings might be due to the gender-specific patterns in alcohol consumption: Since women tended to consume less alcohol and had lower variability in alcohol consumption, we might have had insufficient statistical power to detect effects in women in our linear model. Although a non-linear model did not perform substantially better for our data, it is possible that a more complex model is needed to better capture alcohol effects for women.

Studies regarding the effect of alcohol consumption on general obesity suggest that an excessive amount of consumption is associated with increase in anthropometric measures of obesity, whereas frequent, but light consumption is associated with favorable effects, especially within the context of a “Mediterranean Diet” [33, 34]. However, evidence is far from conclusive, and in the large, population-based UK Biobank study, there was no association between alcohol consumption and anthropometric measurements of body size [35].

Mechanistically, chronic alcohol exposure disrupts muscle growth by dysregulation of the mTORC1 signaling pathway, a central driver of muscle protein synthesis. This includes altered phosphorylation and reduced expression of central intermediates like IRS-1, Akt, and 4E-BP1, as well as upregulation of IGF binding protein-1 and myostatin, all contributing to suppressed anabolic activity [36]. Moreover, catabolic signaling is increased through activation of the Ubiquitin-proteasome pathway and potentially the autophagic-lysosomal system [36]. Regarding myosteatosis, alcohol intake impairs fat oxidation by disrupting mitochondrial function and reducing the activity of enzymes like AMPK and carnitine palmitoyltransferase 1 (CPT1). Due to this impaired oxidation, food intake is less likely to be used for energy production, but rather stored as adipose tissue [37].

### Protein, amino acids, and muscle composition

There is controversy regarding the role of high-or low protein diets for general health. While diets high in protein at the expense of carbohydrates and fats have been found to promote weight loss and reduction in hepatic fat [38], protein-rich diets can also increase the risk of renal hypertension and kidney dysfunction in at-risk populations [39]. Moreover, the source of protein seems to play a major role for its cardiometabolic effects. For example, higher total or animal protein intake increased risk for incident diabetes in a population-based cohort with 10y follow-up [40]. Higher animal protein, but not vegetable protein was associated with increased CVD and total mortality in the Rotterdam Study, and a meta-analysis [41].

Nevertheless, the protective effect of protein rich diets on sarcopenia in older adults is well established [42]. With aging, the anabolic response towards dietary protein intake is lowered, and a higher amount of dietary protein is needed to provide the same level of muscle protein synthesis. A recent cross-sectional analysis of the Japanese National Health and Nutrition Survey on individuals > 60 years showed that participants with high dietary protein intake had higher appendicular muscle mass [43]. In middle-aged adults from the US-based NHANES survey, higher dietary protein intake was associated with increased lean mass and handgrip strength [44]. In our analyses, we did not find an association of dietary protein intake with muscle size as defined by imaging-derived muscle area, but rather an association with decreased fatty infiltration of muscle tissue, indicating an association with muscle quality rather than quantity. This is supported by a study in postmenopausal women, where increased protein intake was associated with increased muscle quality (defined as muscle strength divided by muscle mass), independent of essential amino acid intake [45]. On the other hand, higher intake of BCAA was associated with increased skeletal muscle mass index after adjustment for total energy intake in a large cross-sectional population-based study [46]. Regarding the clinical relevance of the estimated effect for muscle fat, increased protein intake would offset one year of aging, and would almost offset one unit increase in BMI.

Leucine, and to a lesser extent methionine, is implicated in the activation of the mTORC1 pathway which is important for muscle protein synthesis [47], and leucine supplementation has been found to be effective in ameliorating sarcopenia and sarcopenia progression in older adults [48]. In a longitudinal study on trajectories of body composition, increased dietary leucine intake was associated with a decelerated loss of lean body mass in older (> 65 years) individuals over 6 years of follow-up [49]. On the other hand, the Health, Aging, and Body Composition study (mean age 73 years) found no effect of dietary protein, or leucine, intake on thigh muscle area over 5 years of follow-up [50].

We found that an increased proportion of valine and tryptophan to total protein consumed was associated with lower muscle fat, but only in women. Previous evidence regarding the effects of valine intake on body composition is inconsistent. For example, valine supplementation was associated with increased weight gain, lipid accumulation in mice, but there seem to be interaction effects with the overall amount of calories consumed, and the proportion of fat and protein contained in the diet [51]. Moreover amino acid antagonism has to be taken into account: since all three BCAA share the same degradation pathway with branched-chain keto dehydrogenase as the main catabolic enzyme, increased leucine uptake will lead to higher enzymatic activity and accelerated breakdown of isoleucine and valine as well.

Tryptophan is a key player in the brain-gut axis, and affects satiety signaling as a precursor molecule of 5-hydroxytryptamine (serotonine). Circulating peripheral serotonine has been found to be lower in women with morbid obesity compared to women with regular weight and serotonine levels correlated negatively with inflammation markers IL-1 and TNFalpha [52]. Another study found associations between higher serotonine levels and lower body weight and fat mass in men only [53]. Moreover, how dietary tryptophan translates to circulating serotonin is not exactly quantified, since this complex process is influenced by many factors, such as plasma availability of tryptophan, and levels of large neutral amino acids, as well as immune activation [54]. Sex differences in tryptophan metabolism have been described, as women exhibited higher levels of free plasma tryptophan after tryptophan supplementation compared to men [55]. However, further research is needed to investigate sex-specific effects of dietary intake of specific amino acids. Besides potential biological differences in metabolism, also dietary patterns including meal frequency, amount of calories consumed and sources of protein (e.g. plant- vs. animal based) significantly differ according to sex and gender [56], which makes it more difficult to disentangle individual effects of amino acids.

In our analyses, an increased proportion of basic amino acids, i.e. histidine (in women) and lysine (in men) to total protein was associated with increased muscle area. Histidine supplementation has been shown to decrease fat mass in women with obesity [57], and, as a precursor of the dipeptide carnosine, histidine is essential for skeletal muscle function [58]. In an animal model of sarcopenia, lysine supplementation decreased the loss of muscle mass in male mice [59]. Although biologically plausible, our results have to be viewed with caution, since overall protein intake was not associated with muscle area at all. We furthermore have to note that in our data there was no recording of dietary supplements use, resulting in a potential underestimation of actual amino acid intake. Future investigations should also consider the role of dietary intake of non-essential amino acids.

In our sample, individual amino acid intake recommendations were on average exceeded by more than 200%. Still, habitual daily intake for both women and men was comparable to, or even lower than values reported from the EPIC database, which is based on > 500,000 Europeans [60]. The official recommendations have been debated for being too low, especially for older individuals [61]. For example, dietary leucine requirements in individuals older than 60 years were shown to be more than double the recommendations [62]. Thus, although the given thresholds of intake recommendation could be too low, our findings show that dietary amino acid patterns might be relevant for muscle composition even in the absence of major deficiency.

It is important to note the methodological differences between our and previous studies. Primarily, most prior studies evaluate muscle mass and composition by bioimpedance, DXA, or computed tomography, as opposed to MRI used in our study. Moreover, methods of nutritional assessment differ widely between studies. Furthermore, in our analysis, we considered protein composition by evaluating single amino acids as percentage of total protein intake, as opposed to absolute amino acid intake, whereas many studies analyzing the impact of single amino acids use isocaloric substitution of protein by carbohydrates. Some of the discrepancies in findings might be due to these methodological differences.

Our study has several limitations. First, although the sample stems from a population-based cohort, generalizability to the general population is hampered by the usual limitations. Individuals excluded due to missing nutrition data had on average larger body size and worse lipid profile, and we might hypothesize that these individuals had an unhealthier diet which they were unwilling to disclose, leading to a selection bias in the sample [63]. Furthermore when translating our findings to current research, it has to be considered that dietary patterns may have changed compared to 2013–2014 when our data were collected. In future research, also the role of nutrient sources in relation to muscle health should be investigated. Second, the rather limited sample size prohibited us from conducting further subgroup analyses, and from identifying effects with smaller sizes. Previous research on the relationships between nutrition and muscle characteristics has shown that there are complex dependencies between diet composition, obesity status, and physical activity, which need to be further investigated, preferably with more granular data on physical activity. Third, the cross-sectional nature of our data prevents any temporal or causal conclusions, and although it seems more likely that nutrition influences muscle composition than the other way around, we cannot conclusively show that.

Self-reported dietary assessment methods such as the FFQ and 24 h recall lists have inherent limitations. They are affected by recall bias and systematic underreporting, for example due to social desirability bias [64]. To improve validity, we combined the FFQ with repeated 24-h recalls, which provides a more robust estimate of habitual nutrient intake than either method alone. Nonetheless, some degree of misclassification will have occurred. From a statistical perspective, we can assume that this misclassification is non-differential with respect to the analyzed outcomes of muscle fat and area, which would lead to attenuated associations that are biased towards the Null hypothesis. Therefore, while our results should be interpreted with caution, it is possible that the true associations between habitual dietary intake and muscle fat and area are stronger than those observed.

In conclusion, habitual dietary intake of alcohol and protein were associated with muscle fat, but not size. This beneficial effect of nutrition on muscle quality must be further investigated to evaluate its potential to inform personalized, diet-based interventions on muscle health.

## Supplementary Information

Supplementary Material 1.

## Data Availability

The datasets analyzed during the current study are not publicly available due to national data protection laws, since the informed consent given by KORA study participants does not cover data posting in public databases. Data are available upon request by means of a project agreement from KORA. Requests should be sent to kora.passt@helmholtz-munich.de and are subject to approval by the KORA Board. Analysis codes are available from the authors upon reasonable request.
