# Supplementary material for: Association of habitual diet with skeletal muscle composition in a cross-sectional, population-based imaging study
Source: Nutr J. 2025 Sep 23;24:139. doi: 10.1186/s12937-025-01222-5 (PMC12455845; doi:10.1186/s12937-025-01222-5)
Supplement: Supplementary file 1 — Supplementary Material 1. [file 12937_2025_1222_MOESM1_ESM.docx]

Supplementary Material to

**Association of habitual diet with skeletal muscle composition in a cross-sectional, population-based imaging study**

Susanne Rospleszcz^1,2,3^, Theresa Burger^3,4^, Nuha Shugaa Addin^2,3,5^, Lena S. Kiefer^6,7^, Thierno D. Diallo^1^, Nina Wawro^3,10^, Christopher L. Schlett^1^, Fabian Bamberg^1^, Annette Peters^2,3,8,9^, Kurt Gedrich^4^, Jakob Linseisen^10^

^1^Department of Diagnostic and Interventional Radiology, University Medical Center Freiburg, Faculty of Medicine, University of Freiburg, Freiburg, Germany

^2^Chair of Epidemiology, Institute for Medical Information Processing, Biometry, and Epidemiology (IBE), Medical Faculty, Ludwig-Maximilians-Universität (LMU), München, Germany.

^3^Institute of Epidemiology, Helmholtz Zentrum München, German Research Center for Environmental Health, Neuherberg, Germany

^4^Technical University of Munich, ZIEL - Institute for Food & Health, Research Group Public Health Nutrition, Freising, Germany

^5^Pettenkofer School of Public Health, Munich

^6^Department of Diagnostic and Interventional Radiology, Eberhard Karls University of Tuebingen, Tuebingen, Germany

^7^Department of Nuclear Medicine and Clinical Molecular Imaging, Eberhard Karls University of Tuebingen, Tuebingen, Germany

^8^German Center for Diabetes Research (DZD), 85764 Neuherberg, Germany.

^9^German Centre for Cardiovascular Research (DZHK e.V.), Partner Site Munich Heart Alliance, 80802 München, Germany

^10^Epidemiology, University of Augsburg, University Hospital Augsburg, Augsburg, Germany

**Supplementary Texts**

Supplementary Text 1: Details of the assessment of dietary intake.

Participants filled out the Food Frequency Questionnaire (FFQ) electronically at home, within a week after the visit at the study center. They were given a phone call reminder if they did not return the questionnaire within a week, and a second reminder after two weeks. The FFQ covered the time frame of the past year (12 months). Participants reported usual consumption frequency and usual portion size. Frequency was assessed categorically as “never”, “ ≤1x per month”, “2-3x per month”, “1-2x per week”, “3-4x per week” “5-6x per week”, ”1x per day”, ”2x per day”, ”3x per day”, “>3x per day”. Portion size was assessed using pictograms.

The first 24-h recall list was filled out electronically at the study center visit. Participants were asked to complete two additional 24-h recall lists (one on a weekday, and one on a weekend) at home within three months after their visit at the study center.

**Supplementary Tables**

Supplementary Table 1: Difference in baseline characteristics between individuals excluded from the final sample and those included in the final sample.

|  | Excluded | Included | p-value |
| --- | --- | --- | --- |
|  | N = 105 | N = 294 |  |
| **Demographics** |  |  |  |
| Age, years | 56.0 ± 9.7 | 56.5 ± 9.0 | 0.651 |
| Male sex | 68 (64.8%) | 162 (55.1%) | 0.109 |
| **Anthropometrics** |  |  |  |
| Height, cm | 172.7 ± 9.6 | 171.2 ± 9.8 | 0.183 |
| Weight, kg | 86.9 ± 17.7 | 81.6 ± 15.9 | 0.005 |
| BMI, kg/m^2^ | 29.1 ± 5.1 | 27.8 ± 4.8 | 0.024 |
| Waist circumference, cm | 101.8 ± 14.8 | 97.5 ± 14.0 | 0.008 |
| Hip circumference, cm | 108.5 ± 9.7 | 106.5 ± 8.9 | 0.046 |
| Waist-to-Hip Ratio | 0.936 ± 0.088 | 0.914 ± 0.0.89 | 0.027 |
| **Lifestyle factors** |  |  |  |
| Smoking behaviour |  |  |  |
| Neversmoker | 36 (34.3%) | 109 (37.1%) | 0.814 |
| ex-smoker | 46 (43.8%) | 128 (43.5%) |  |
| regular smoker | 23 (21.9%) | 57 (19.4%) |  |
| Physical activity |  |  | 0.644 |
| regularly 2h/w or more | 27 (25.7%) | 87 (29.6%) |  |
| regularly 1h/w | 30 (28.6%) | 93 (31.6%) |  |
| sporadically | 16 (15.2%) | 41 (13.9%) |  |
| inactive | 32 (30.5%) | 73 (24.8%) |  |
| **Blood pressure** |  |  |  |
| Systolic Blood Pressure, mmHg | 122.0 ± 17.7 | 120.2 ± 16.4 | 0.357 |
| Diastolic Blood Pressure, mmHg | 76.2 ± 10.1 | 75.0 ± 10.0 | 0.273 |
| Hypertension | 32 (30.5%) | 104 (35.4%) | 0.430 |
| Antihypertensive medication | 22 (21.0%) | 80 (27.2%) | 0.258 |
| **Diabetes-related** |  |  |  |
| Glycemia |  |  |  |
| Normogylcemia | 57 (54.3%) | 185 (62.9%) | 0.254 |
| Prediabetes | 30 (28.6%) | 73 (24.8%) |  |
| Diabetes | 18 (17.1%) | 36 (12.2%) |  |
| HbA1c, % | 5.7 ± 1.0 | 5.5 ± 0.6 | 0.050 |
| Fasting glucose, mg/dL | 107.5 ± 32.8 | 103.1 ± 17.6 | 0.089 |
| 2-h glucose, mg/dL^#^ | 117.4 ± 39.8 | 111.9 ± 41.2 | 0.264 |
| Fasting insulin, μU/mL | 12.1 ± 8.3 | 11.1 ± 7.2 | 0.246 |
| 2-h insulin, μU/mL^#^ | 73.1 ± 66.5 | 64.3 ± 66.4 | 0.272 |
| Glucose-lowering medication | 9 (8.6%) | 23 (7.8%) | 0.974 |
| **Lipid profile** |  |  |  |
| Total Cholesterol, mg/dL | 218.4 ± 35.6 | 217.7 ± 36.6 | 0.877 |
| HDL Cholesterol, mg/dL | 59.0 ± 16.8 | 62.9 ± 17.9 | 0.051 |
| LDL Cholesterol, mg/dL | 140.0 ± 31.0 | 139.4 ± 33.6 | 0.876 |
| Triglycerides, mg/dL | 147.0 ± 97.2 | 126.2 ± 79.5 | 0.031 |
| Lipid-lowering medication | 10 (9.5%) | 33 (11.2%) | 0.765 |
| **Inflammation** |  |  |  |
| Uric Acid, mg/dL | 5.7 ± 1.5 | 5.6 ± 1.5 | 0.556 |
| hsCRP, mg/L | 2.3 ± 2.7 | 2.4 ± 3.5 | 0.895 |

Data are means and standard deviation for continuous variables, and counts and percentages for categorical values. P-values from t-test and Χ^2^ test, respectively. ^#^based on n=363 with Oral Glucose Tolerance Test data.

Supplementary Table 2: Habitual intake of selected foods and nutrients.

| **Food/Nutrient** | **unit** | **Whole sample** | **Women** | **Men** |
| --- | --- | --- | --- | --- |
|  |  | N = 294 | N = 132 | N = 162 |
| Vegetables | g/d | 166.5 ± 60.8 | 191.8 ± 68.3 | 145.9 ± 44.4 |
| Fruits and Nuts | g/d | 146.1 ± 74.1 | 160.0 ± 72.3 | 134.8 ± 73.9 |
| Monosaccharides | g/d | 320.0 ± 138.5 | 287.9 ± 852.8 | 346.1 ± 165.7 |
| Disaccharides | g/d | 623.0 ± 224.4 | 574.4 ± 206.0 | 662.7 ± 231.6 |
| Saturated Fatty Acids | g/d | 34.7 ± 7.7 | 30.3 ± 6.2 | 38.4 ± 6.8 |
| Monounsaturated Fatty Acids | g/d | 27.3 ± 6.3 | 23.2 ± 4.9 | 30.5 ± 5.3 |
| Polyunsaturated Fatty Acids | g/d | 9.9 ± 2.6 | 8.7 ± 2.0 | 10.9 ± 2.6 |
| Butter | g/d | 13.3 ± 6.5 | 10.6 ± 4.9 | 15.4 ± 6.9 |
| Oils, plant-based | g/d | 5.8 ± 3.2 | 6.0 ± 3.0 | 5.7 ± 3.3 |
| Beef | g/d | 8.7 ± 5.1 | 6.3 ± 4.1 | 10.7 ± 5.0 |
| Pork | g/d | 20.8 ± 10.4 | 15.2 ± 5.7 | 25.3 ± 11.2 |
| Poultry | g/d | 15.5 ± 7.6 | 14.3 ± 8.0 | 16.4 ± 7.2 |
| Fish, Shellfish | g/d | 22.3 ± 14.8 | 18.5 ± 9.8 | 25.3 ± 17.3 |
| Wine | g/d | 41.5 ± 52.3 | 33.9 ± 43.6 | 47.7 ± 57.8 |
| Beer | g/d | 173.1 ± 228.6 | 19.2 ± 42.2 | 298.6 ± 241.7 |
| Spirits | g/d | 0.6 ± 1.1 | 0.3 ± 0.3 | 0.9 ± 1.4 |
| Cocktails, punch | g/d | 20.6 ± 38.3 | 15.3 ± 26.2 | 25.0 ± 45.5 |
| Fiber | g/d | 16.5 ± 4.4 | 16.3 ± 4.3 | 16.6 ± 4.6 |
| Sodium | mg/d | 2114.5 ± 563.1 | 1776.9 ± 424.7 | 2389.5 ± 510.7 |

Data are presented as means and standard deviation.

Supplementary Table 3: Association of habitual energy-providing nutrient intake with MRI-derived muscle fat and area – effect estimates for all variables in the model.

|  | Outcome Muscle Fat, % | | | Outcome Muscle Area, cm^2^ | | |
| --- | --- | --- | --- | --- | --- | --- |
|  | β | 95%-CI | p-value | β | 95%-CI | p-value |
| **Carbohydrates, per 1% of total energy intake** | | | | | | |
| **All** | | | | | | |
| Carbohydrates | -0.06 | [-0.20, 0.07] | 0.356 | -0.11 | [-0.47, 0.24] | 0.520 |
| Age, years | 0.34 | [0.28, 0.41] | 0.000 | -0.49 | [-0.66, -0.33] | 0.000 |
| BMI, kg/m^2^ | 0.34 | [0.22, 0.46] | 0.000 | 1.24 | [0.93, 1.54] | 0.000 |
| Male sex | -4.34 | [-5.43, -3.25] | 0.000 | 27.73 | [24.95, 30.51] | 0.000 |
| Physical Activity: regularly 1h/w | -0.04 | [-1.41, 1.33] | 0.956 | -1.88 | [-5.39, 1.63] | 0.292 |
| Physical Activity: sporadically | 0.41 | [-1.38, 2.21] | 0.651 | -5.04 | [-9.62, -0.45] | 0.031 |
| Physical Activity: inactive | 0.98 | [-0.55, 2.51] | 0.207 | -2.21 | [-6.12, 1.71] | 0.268 |
| Glycemia: Prediabetes | -0.33 | [-1.71, 1.04] | 0.633 | 1.79 | [-1.72, 5.30] | 0.316 |
| Glycemia: Diabetes | 1.41 | [-0.43, 3.26] | 0.133 | -0.20 | [-4.91, 4.51] | 0.933 |
| **Women** | | | | | | |
| Carbohydrates | -0.01 | [-0.26, 0.23] | 0.911 | -0.05 | [-0.56, 0.47] | 0.859 |
| Age, years | 0.44 | [0.33, 0.55] | 0.000 | -0.37 | [-0.59, -0.15] | 0.001 |
| BMI, kg/m^2^ | 0.31 | [0.12, 0.49] | 0.001 | 1.02 | [0.64, 1.39] | 0.000 |
| Physical Activity: regularly 1h/w | -0.25 | [-2.38, 1.87] | 0.814 | -1.96 | [-6.36, 2.44] | 0.380 |
| Physical Activity: sporadically | 1.44 | [-1.39, 4.27] | 0.317 | -2.56 | [-8.43, 3.31] | 0.389 |
| Physical Activity: inactive | 1.60 | [-1.16, 4.36] | 0.252 | -0.64 | [-6.36, 5.07] | 0.824 |
| Glycemia: Prediabetes | -1.35 | [-3.85, 1.16] | 0.289 | 5.90 | [0.71, 11.09] | 0.026 |
| Glycemia: Diabetes | 3.71 | [0.49, 6.93] | 0.024 | 3.15 | [-3.52, 9.81] | 0.352 |
| **Men** | | | | | | |
| Carbohydrates | -0.11 | [-0.27, 0.05] | 0.161 | -0.18 | [-0.67, 0.31] | 0.465 |
| Age, years | 0.29 | [0.21, 0.37] | 0.000 | -0.57 | [-0.81, -0.33] | 0.000 |
| BMI, kg/m^2^ | 0.37 | [0.20, 0.53] | 0.000 | 1.33 | [0.82, 1.84] | 0.000 |
| Physical Activity: regularly 1h/w | 0.07 | [-1.70, 1.83] | 0.941 | -1.47 | [-6.91, 3.98] | 0.595 |
| Physical Activity: sporadically | -0.37 | [-2.64, 1.90] | 0.746 | -7.69 | [-14.69, -0.69] | 0.032 |
| Physical Activity: inactive | 0.53 | [-1.24, 2.29] | 0.555 | -3.61 | [-9.05, 1.83] | 0.192 |
| Glycemia: Prediabetes | 0.05 | [-1.52, 1.62] | 0.952 | -0.90 | [-5.74, 3.94] | 0.715 |
| Glycemia: Diabetes | 0.35 | [-1.82, 2.52] | 0.751 | -1.78 | [-8.48, 4.92] | 0.601 |
| **Fat, per 1% of total energy intake** | | | | | | |
| **All** | | | | | | |
| Fat | -0.1 | [-0.27, 0.06] | 0.209 | 0.11 | [-0.30, 0.53] | 0.589 |
| Age, years | 0.35 | [0.29, 0.41] | 0.000 | -0.49 | [-0.66, -0.33] | 0.000 |
| BMI, kg/m^2^ | 0.34 | [0.22, 0.46] | 0.000 | 1.24 | [0.93, 1.54] | 0.000 |
| Male sex | -4.43 | [-5.54, -3.33] | 0.000 | 28.03 | [25.20, 30.85] | 0.000 |
| Physical Activity: regularly 1h/w | 0.04 | [-1.33, 1.41] | 0.958 | -1.86 | [-5.37, 1.65] | 0.297 |
| Physical Activity: sporadically | 0.7 | [-1.11, 2.52] | 0.446 | -5.12 | [-9.76, -0.48] | 0.031 |
| Physical Activity: inactive | 1.28 | [-0.25, 2.82] | 0.101 | -2.20 | [-6.13, 1.73] | 0.272 |
| Glycemia: Prediabetes | -0.19 | [-1.56, 1.17] | 0.780 | 1.86 | [-1.63, 5.36] | 0.294 |
| Glycemia: Diabetes | 1.64 | [-0.21, 3.48] | 0.082 | -0.22 | [-4.95, 4.51] | 0.927 |
| **Women** | | | | | | |
| Fat | -0.13 | [-0.41, 0.14] | 0.335 | -0.4 | [-0.96, 0.17] | 0.167 |
| Age, years | 0.45 | [0.34, 0.55] | 0.000 | -0.35 | [-0.57, -0.12] | 0.003 |
| BMI, kg/m^2^ | 0.31 | [0.13, 0.49] | 0.001 | 1.03 | [0.65, 1.41] | 0.000 |
| Physical Activity: regularly 1h/w | -0.29 | [-2.41, 1.82] | 0.784 | -2.08 | [-6.44, 2.28] | 0.347 |
| Physical Activity: sporadically | 1.65 | [-1.21, 4.50] | 0.256 | -1.95 | [-7.84, 3.94] | 0.514 |
| Physical Activity: inactive | 1.93 | [-0.83, 4.69] | 0.168 | 0.34 | [-5.36, 6.04] | 0.906 |
| Glycemia: Prediabetes | -1.21 | [-3.70, 1.28] | 0.338 | 6.32 | [1.18, 11.45] | 0.016 |
| Glycemia: Diabetes | 3.72 | [0.51, 6.93] | 0.023 | 3.18 | [-3.44, 9.80] | 0.344 |
| **Men** | | | | | | |
| Fat | -0.09 | [-0.28, 0.11] | 0.380 | 0.44 | [-0.16, 1.04] | 0.153 |
| Age, years | 0.29 | [0.22, 0.37] | 0.000 | -0.56 | [-0.80, -0.33] | 0.000 |
| BMI, kg/m^2^ | 0.37 | [0.21, 0.54] | 0.000 | 1.32 | [0.81, 1.82] | 0.000 |
| Physical Activity: regularly 1h/w | 0.34 | [-1.43, 2.10] | 0.706 | -1.66 | [-7.06, 3.74] | 0.545 |
| Physical Activity: sporadically | 0.13 | [-2.15, 2.41] | 0.910 | -8.22 | [-15.20, -1.24] | 0.021 |
| Physical Activity: inactive | 0.88 | [-0.90, 2.65] | 0.330 | -3.97 | [-9.39, 1.45] | 0.150 |
| Glycemia: Prediabetes | 0.22 | [-1.35, 1.78] | 0.782 | -0.86 | [-5.65, 3.92] | 0.722 |
| Glycemia: Diabetes | 0.76 | [-1.45, 2.97] | 0.496 | -2.47 | [-9.22, 4.28] | 0.471 |
| **Protein, per 1% of total energy intake** | | | | | | |
| **All** | | | | | | |
| Protein | -0.34 | [-0.69, 0.00] | 0.052 | -0.11 | [-1.00, 0.77] | 0.801 |
| Age, years | 0.34 | [0.28, 0.40] | 0.000 | -0.49 | [-0.66, -0.33] | 0.000 |
| BMI, kg/m^2^ | 0.38 | [0.25, 0.50] | 0.000 | 1.25 | [0.93, 1.57] | 0.000 |
| Male sex | -4.59 | [-5.71, -3.47] | 0.000 | 27.74 | [24.87, 30.62] | 0.000 |
| Physical Activity: regularly 1h/w | 0.03 | [-1.33, 1.39] | 0.966 | -1.81 | [-5.31, 1.70] | 0.312 |
| Physical Activity: sporadically | 0.32 | [-1.46, 2.11] | 0.724 | -4.95 | [-9.54, -0.36] | 0.035 |
| Physical Activity: inactive | 1.03 | [-0.48, 2.53] | 0.180 | -2.02 | [-5.89, 1.84] | 0.304 |
| Glycemia: Prediabetes | -0.27 | [-1.63, 1.09] | 0.696 | 1.93 | [-1.56, 5.41] | 0.278 |
| Glycemia: Diabetes | 1.48 | [-0.34, 3.31] | 0.111 | -0.06 | [-4.76, 4.63] | 0.979 |
| **Women** | | | | | | |
| Protein | -0.23 | [-0.78, 0.31] | 0.403 | -0.21 | [-1.35, 0.92] | 0.710 |
| Age, years | 0.43 | [0.32, 0.54] | 0.000 | -0.37 | [-0.60, -0.15] | 0.001 |
| BMI, kg/m^2^ | 0.33 | [0.14, 0.53] | 0.001 | 1.04 | [0.64, 1.45] | 0.000 |
| Physical Activity: regularly 1h/w | -0.22 | [-2.34, 1.90] | 0.836 | -1.94 | [-6.34, 2.45] | 0.383 |
| Physical Activity: sporadically | 1.26 | [-1.60, 4.11] | 0.384 | -2.74 | [-8.66, 3.19] | 0.362 |
| Physical Activity: inactive | 1.64 | [-1.05, 4.34] | 0.230 | -0.53 | [-6.13, 5.06] | 0.851 |
| Glycemia: Prediabetes | -1.34 | [-3.82, 1.14] | 0.286 | 5.95 | [0.80, 11.09] | 0.024 |
| Glycemia: Diabetes | 3.50 | [0.26, 6.75] | 0.035 | 2.96 | [-3.78, 9.70] | 0.386 |
| **Men** | | | | | | |
| Protein | -0.31 | [-0.76, 0.13] | 0.169 | 0.16 | [-1.22, 1.53] | 0.823 |
| Age, years | 0.28 | [0.21, 0.36] | 0.000 | -0.56 | [-0.80, -0.32] | 0.000 |
| BMI, kg/m^2^ | 0.40 | [0.23, 0.57] | 0.000 | 1.32 | [0.80, 1.84] | 0.000 |
| Physical Activity: regularly 1h/w | 0.25 | [-1.49, 2.00] | 0.775 | -1.19 | [-6.59, 4.21] | 0.664 |
| Physical Activity: sporadically | -0.17 | [-2.41, 2.06] | 0.878 | -7.16 | [-14.06, -0.25] | 0.042 |
| Physical Activity: inactive | 0.64 | [-1.11, 2.38] | 0.472 | -3.22 | [-8.61, 2.17] | 0.239 |
| Glycemia: Prediabetes | 0.18 | [-1.37, 1.74] | 0.816 | -0.68 | [-5.49, 4.13] | 0.780 |
| Glycemia: Diabetes | 0.72 | [-1.45, 2.88] | 0.514 | -1.52 | [-8.21, 5.16] | 0.653 |
| **Alcohol, per 1% of total energy intake** | | | | | | |
| **All** | | | | | | |
| Alcohol | 0.31 | [0.14, 0.48] | <0.001 | 0.08 | [-0.37, 0.53] | 0.721 |
| Age | 0.34 | [0.27, 0.40] | 0.000 | -0.49 | [-0.66, -0.33] | 0.000 |
| BMI, kg/m^2^ | 0.38 | [0.26, 0.50] | 0.000 | 1.25 | [0.94, 1.56] | 0.000 |
| Male sex | -5.45 | [-6.69, -4.22] | 0.000 | 27.55 | [24.32, 30.77] | 0.000 |
| Physical Activity: regularly 1h/w | -0.05 | [-1.39, 1.30] | 0.947 | -1.83 | [-5.33, 1.68] | 0.306 |
| Physical Activity: sporadically | 0.51 | [-1.24, 2.26] | 0.567 | -4.89 | [-9.45, -0.32] | 0.036 |
| Physical Activity: inactive | 0.98 | [-0.50, 2.46] | 0.193 | -2.03 | [-5.89, 1.84] | 0.303 |
| Glycemia: Prediabetes | -0.49 | [-1.83, 0.85] | 0.474 | 1.87 | [-1.63, 5.37] | 0.294 |
| Glycemia: Diabetes | 1.53 | [-0.27, 3.33] | 0.096 | -0.05 | [-4.74, 4.64] | 0.983 |
| **Women**^#^ | | | | | | |
| Alcohol | -0.01 | [-0.49, 0.47] | 0.971 | 0.61 | [-0.41, 1.63] | 0.240 |
| Age, years | 0.44 | [0.34, 0.54] | 0.000 | -0.37 | [-0.58, -0.15] | 0.001 |
| BMI, kg/m^2^ | 0.30 | [0.11, 0.48] | 0.002 | 1.09 | [0.70, 1.47] | 0.000 |
| Physical Activity: regularly 1h/w | -0.20 | [-2.21, 1.82] | 0.847 | -1.71 | [-5.96, 2.53] | 0.426 |
| Physical Activity: sporadically | 1.63 | [-1.08, 4.34] | 0.237 | -1.78 | [-7.50, 3.94] | 0.539 |
| Physical Activity: inactive | 1.08 | [-1.50, 3.66] | 0.410 | -1.24 | [-6.68, 4.20] | 0.652 |
| Glycemia: Prediabetes | -1.23 | [-3.60, 1.14] | 0.305 | 5.77 | [0.77, 10.77] | 0.024 |
| Glycemia: Diabetes | 2.25 | [-0.88, 5.39] | 0.158 | 0.97 | [-5.65, 7.59] | 0.772 |
| **Men** | | | | | | |
| Alcohol | 0.28 | [0.10, 0.45] | 0.002 | -0.17 | [-0.73, 0.39] | 0.545 |
| Age, years | 0.27 | [0.20, 0.35] | 0.000 | -0.55 | [-0.79, -0.31] | 0.000 |
| BMI, kg/m^2^ | 0.40 | [0.24, 0.56] | 0.000 | 1.31 | [0.80, 1.83] | 0.000 |
| Physical Activity: regularly 1h/w | 0.10 | [-1.60, 1.81] | 0.904 | -1.10 | [-6.50, 4.30] | 0.689 |
| Physical Activity: sporadically | -0.30 | [-2.48, 1.88] | 0.785 | -7.07 | [-13.96, -0.17] | 0.045 |
| Physical Activity: inactive | 0.55 | [-1.15, 2.25] | 0.521 | -3.16 | [-8.53, 2.22] | 0.248 |
| Glycemia: Prediabetes | -0.05 | [-1.58, 1.48] | 0.948 | -0.54 | [-5.37, 4.29] | 0.826 |
| Glycemia: Diabetes | 0.82 | [-1.28, 2.93] | 0.442 | -1.61 | [-8.27, 5.05] | 0.634 |

Data for the energy-providing nutrients are repeated from main Table 3. # based on N=131 after excluding one outlier.

Supplementary Table 4: Association of habitual energy-providing nutrient intake with MRI-derived muscle fat and area in sensitivity analyses.

|  | Outcome Muscle Fat, % | | | Outcome Muscle Area, cm^2^ | | |
| --- | --- | --- | --- | --- | --- | --- |
|  | β | 95%-CI | p-value | β | 95%-CI | p-value |
| **Carbohydrates, per 1% of total energy intake** | | | | | | |
| **All** | | | | | | |
| main | -0.06 | [-0.20, 0.07] | 0.356 | -0.11 | [-0.47, 0.24] | 0.520 |
| + Smoking | -0.06 | [-0.20, 0.08] | 0.382 | -0.10 | [-0.45, 0.25] | 0.578 |
| WC instead of BMI | -0.05 | [-0.18, 0.08] | 0.439 | -0.09 | [-0.45, 0.26] | 0.598 |
| **Women** | | | | | | |
| main | -0.01 | [-0.26, 0.23] | 0.911 | -0.05 | [-0.56, 0.47] | 0.859 |
| + Smoking | 0.01 | [-0.25, 0.26] | 0.955 | -0.04 | [-0.57, 0.48] | 0.875 |
| WC instead of BMI | 0.03 | [-0.21, 0.26] | 0.825 | 0.01 | [-0.50, 0.53] | 0.959 |
| **Men** | | | | | | |
| main | -0.11 | [-0.27, 0.05] | 0.161 | -0.18 | [-0.67, 0.31] | 0.465 |
| + Smoking | -0.12 | [-0.28, 0.04] | 0.156 | -0.19 | [-0.67, 0.30] | 0.448 |
| WC instead of BMI | -0.11 | [-0.27, 0.04] | 0.148 | -0.19 | [-0.68, 0.30] | 0.449 |
| **Fat, per 1% of total energy intake** | | | | | | |
| **All** | | | | | | |
| main | -0.1 | [-0.27, 0.06] | 0.209 | 0.11 | [-0.30, 0.53] | 0.589 |
| + Smoking | -0.1 | [-0.27, 0.06] | 0.207 | 0.08 | [-0.32, 0.49] | 0.684 |
| WC instead of BMI | -0.1 | [-0.25, 0.06] | 0.223 | 0.16 | [-0.25, 0.58] | 0.437 |
| **Women** | | | | | | |
| main | -0.13 | [-0.41, 0.14] | 0.335 | -0.40 | [-0.96, 0.17] | 0.167 |
| + Smoking | -0.14 | [-0.41, 0.14] | 0.332 | -0.40 | [-0.97, 0.17] | 0.163 |
| WC instead of BMI | -0.16 | [-0.42, 0.09] | 0.207 | -0.43 | [-1.00, 0.13] | 0.134 |
| **Men** | | | | | | |
| main | -0.09 | [-0.28, 0.11] | 0.380 | 0.44 | [-0.16, 1.04] | 0.153 |
| + Smoking | -0.09 | [-0.28, 0.11] | 0.389 | 0.43 | [-0.16, 1.02] | 0.156 |
| WC instead of BMI | -0.06 | [-0.25, 0.13] | 0.548 | 0.53 | [-0.07, 1.13] | 0.085 |
| **Protein, per 1% of total energy intake** | | | | | | |
| **All** | | | | | | |
| main | -0.34 | [-0.69, 0.00] | 0.052 | -0.11 | [-1.00, 0.77] | 0.801 |
| + Smoking | -0.35 | [-0.69, 0.00] | 0.051 | -0.14 | [-1.02, 0.73] | 0.748 |
| WC instead of BMI | -0.36 | [-0.68, -0.04] | 0.029 | 0.14 | [-0.74, 1.02] | 0.759 |
| **Women** | | | | | | |
| main | -0.23 | [-0.78, 0.31] | 0.403 | -0.21 | [-1.35, 0.92] | 0.710 |
| + Smoking | -0.23 | [-0.78, 0.32] | 0.413 | -0.25 | [-1.40, 0.90] | 0.671 |
| WC instead of BMI | -0.34 | [-0.83, 0.16] | 0.181 | -0.04 | [-1.16, 1.07] | 0.942 |
| **Men** | | | | | | |
| main | -0.31 | [-0.76, 0.13] | 0.169 | 0.16 | [-1.22, 1.53] | 0.823 |
| + Smoking | -0.31 | [-0.76, 0.13] | 0.168 | 0.17 | [-1.18, 1.53] | 0.799 |
| WC instead of BMI | -0.29 | [-0.72, 0.14] | 0.187 | 0.33 | [-1.04, 1.71] | 0.631 |
| **Alcohol, per 1% of total energy intake** | | | | | | |
| **All** | | | | | | |
| main | 0.31 | [0.14, 0.48] | <0.001 | 0.08 | [-0.37, 0.53] | 0.721 |
| + Smoking | 0.32 | [0.14, 0.49] | <0.001 | 0.10 | [-0.35, 0.54] | 0.669 |
| WC instead of BMI | 0.28 | [0.12, 0.44] | <0.001 | -0.08 | [-0.52, 0.37] | 0.726 |
| **Women^#^** | | | | | | |
| main | -0.01 | [-0.49, 0.47] | 0.971 | 0.61 | [-0.41, 1.63] | 0.240 |
| + Smoking | -0.08 | [-0.58, 0.42] | 0.760 | 0.66 | [-0.39, 1.71] | 0.217 |
| WC instead of BMI | 0.07 | [-0.37, 0.51] | 0.746 | 0.39 | [-0.62, 1.40] | 0.442 |
| **Men** | | | | | | |
| main | 0.28 | [0.10, 0.45] | 0.002 | -0.17 | [-0.73, 0.39] | 0.545 |
| + Smoking | 0.28 | [0.10, 0.46] | 0.002 | -0.16 | [-0.71, 0.39] | 0.566 |
| WC instead of BMI | 0.25 | [0.08, 0.42] | 0.005 | -0.27 | [-0.83, 0.29] | 0.338 |

Data for the main model are repeated from Table 3 in the manuscript. The main model was adjusted for variables age, sex (for the whole sample), BMI, physical activity (4 categories), and glycemia (normoglycemia/ prediabetes/ diabetes). WC: waist circumference. # based on N=131 after excluding one outlier.

Supplementary Table 5: WHO recommended daily allowance (RDA) of essential amino acids, and percentage of this recommendation attained in the sample.

|  | WHO RDA mg per kg body mass |  | Whole sample | Women | Men | p-value women vs men |
| --- | --- | --- | --- | --- | --- | --- |
|  |  |  | N = 294 | N = 132 | N = 162 |  |
|  |  | % of RDA | | | |  |
| Phenylalanine and Tyrosine | 25 | mean ± sd | 278.5 ± 72.1 | 280.2 ± 81.5 | 277.1 ± 63.7 | 0.709 |
| Phenylalanine and Tyrosine |  | [min, max] | [143.7, 647.6] | [143.7, 647.6] | [168.9, 523.7] |  |
| Valine | 26 | mean ± sd | 186.6 ± 46.8 | 185.8 ± 52.5 | 187.2 ± 41.9 | 0.804 |
| Valine |  | [min, max] | [95.0, 415.5] | [95.0, 415.5] | [114.3, 344.1] |  |
| Tryptophan | 4 | mean ± sd | 255.1 ± 63.9 | 251.4 ± 71.6 | 258.1 ± 57.0 | 0.371 |
| Tryptophan |  | [min, max] | [128.2, 554.6] | [128.2, 554.6] | [155.3, 471.4] |  |
| Threonine | 15 | mean ± sd | 237.8 ± 58.6 | 234.7 ± 64.9 | 240.2 ± 53.0 | 0.424 |
| Threonine |  | [min, max] | [121.4, 513.8] | [121.4, 513.8] | [147.6, 422.7] |  |
| Isoleucine | 20 | mean ± sd | 206.7 ± 51.1 | 205.9 ± 57.0 | 207.4 ± 46.0 | 0.797 |
| Isoleucine |  | [min, max] | [105.3, 445.9] | [105.3, 445.9] | [129.5, 370.5] |  |
| Methionine and Cysteine | 14.5 | mean ± sd | 214.5 ± 52.7 | 211.7 ± 58.4 | 216.8 ± 47.5 | 0.414 |
| Methionine and Cysteine |  | [min, max] | [111.0, 479.3] | [111.0, 479.3] | [133.9, 384.3] |  |
| Histidine | 10 | mean ± sd | 248.8 ± 58.1 | 241.1 ± 62.3 | 255.1 ± 53.9 | 0.040 |
| Histidine |  | [min, max] | [124.1, 504.4] | [124.1, 504.4] | [161.1, 451.5] |  |
| Leucine | 39 | mean ± sd | 176.3 ± 45.2 | 177.1 ± 51.2 | 175.6 ± 39.9 | 0.784 |
| Leucine |  | [min, max] | [91.8, 405.9] | [91.8, 405.9] | [107.4, 320.3] |  |
| Lysine | 30 | mean ± sd | 198.2 ± 48.8 | 196.6 ± 54.4 | 199.5 ± 43.9 | 0.603 |
| Lysine |  | [min, max] | [103.8, 404.3] | [103.8, 404.3] | [122.8, 340.3] |  |

**Supplementary Figures**

Supplementary Figure 1: Correlation of habitual macronutrient intake with muscle area


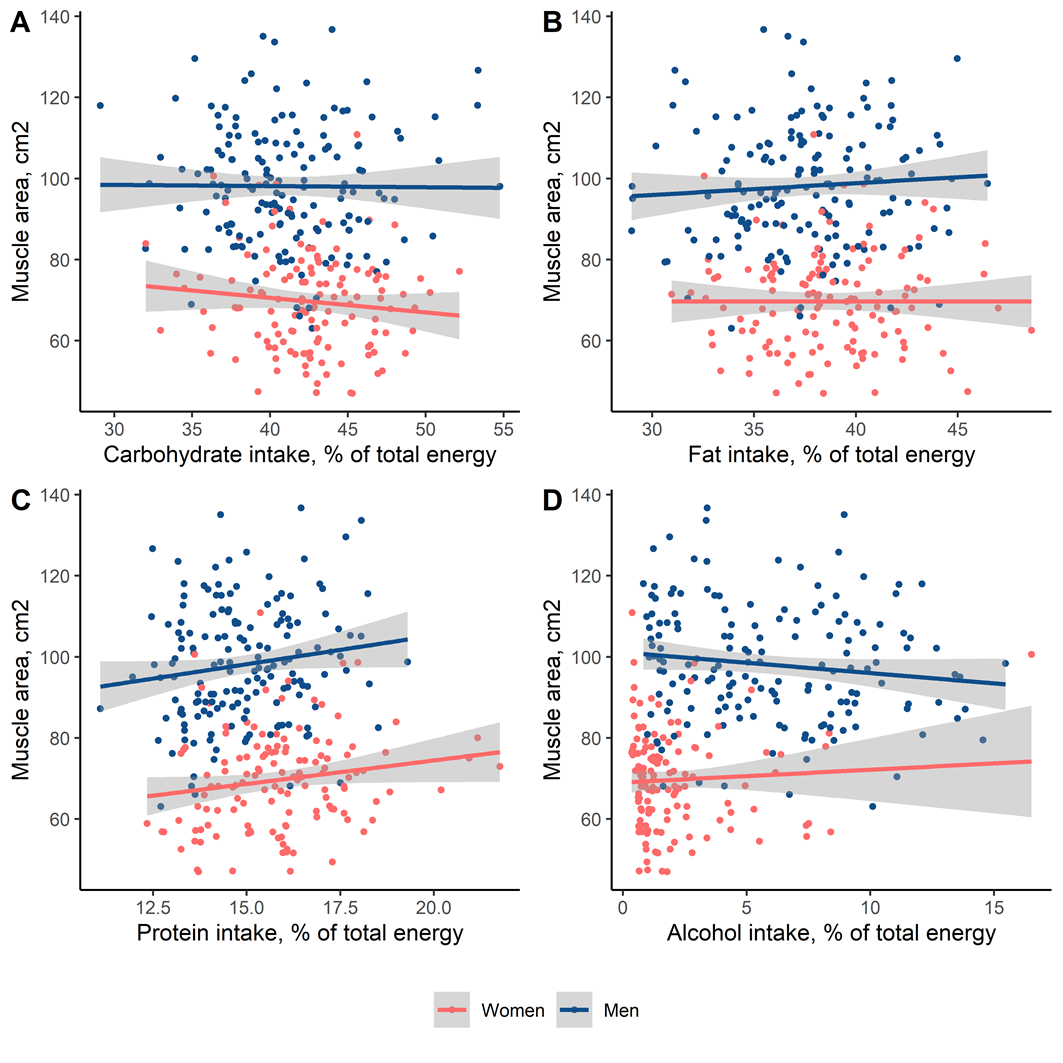


Supplementary Figure 2: Correlation of habitual dietary intake of essential amino acids with muscle area


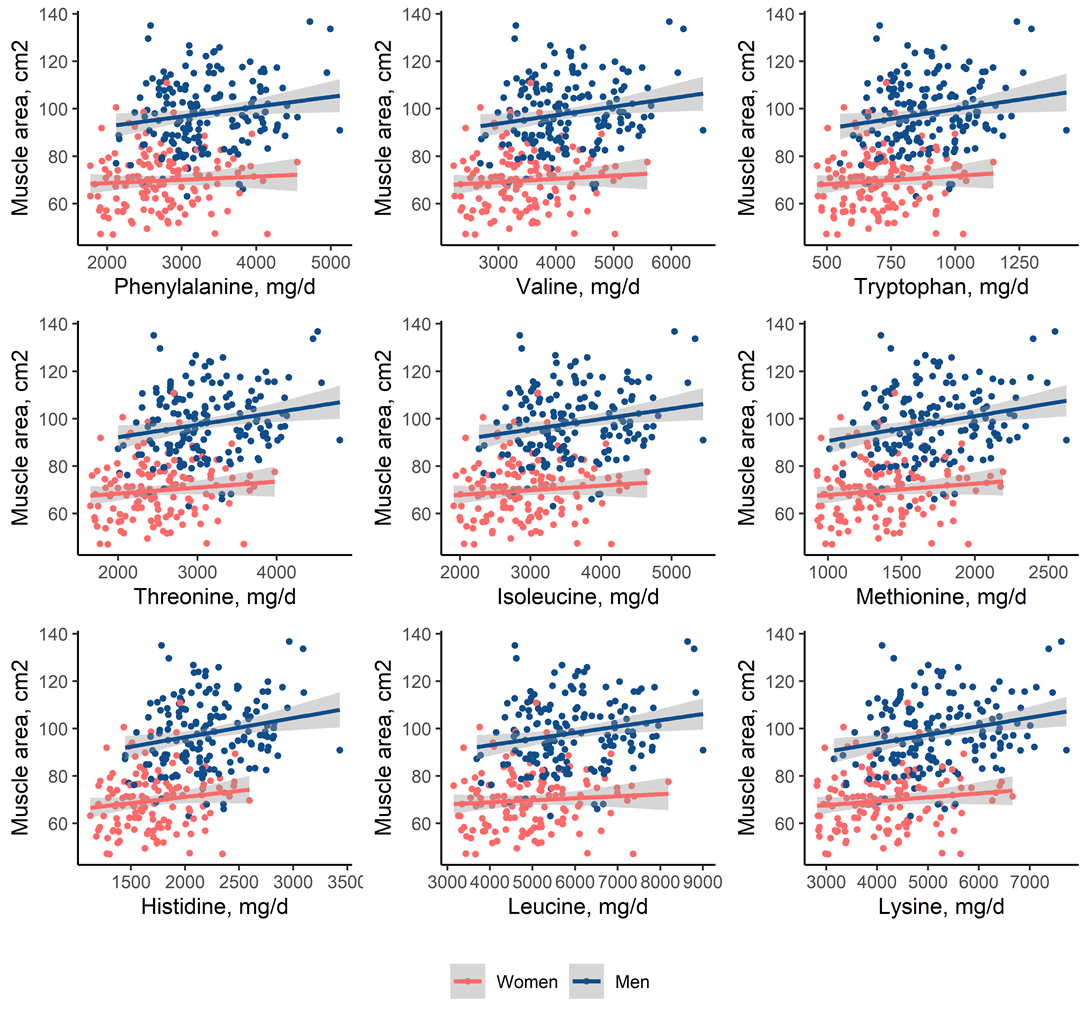


Supplementary Figure 3: Percentage of recommended intake of essential amino acids attained.


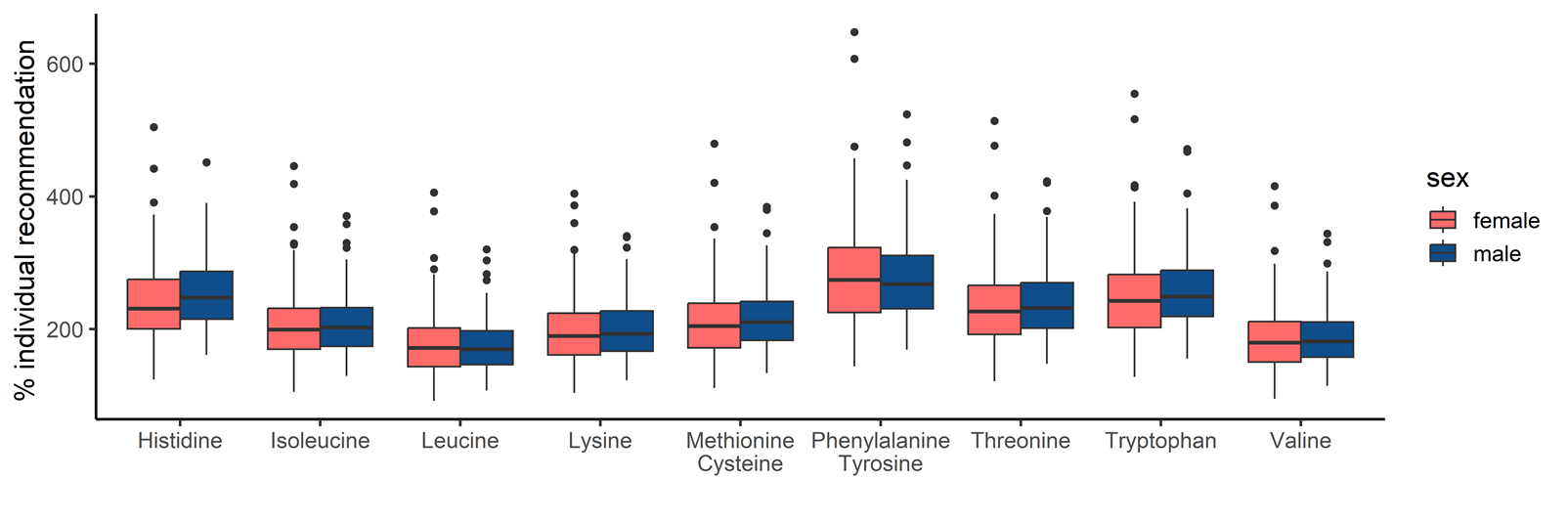


For each amino acid and each participant, individual recommended intake was calculated based on WHO RDA (Supplementary Table 2) and individual body weight. Actual intake was then divided by individual recommended intake to calculate percentage of recommended intake attained.
